# Body mass index at baseline directly predicts new-onset diabetes and to a lesser extent incident cardio-cerebrovascular events, but has a J-shaped relationship to all-cause mortality

**DOI:** 10.1186/s12902-022-01041-3

**Published:** 2022-05-11

**Authors:** Yoon-Jong Bae, Sang-Jun Shin, Hee-Taik Kang

**Affiliations:** 1 Department of Information & Statistics, Chungbuk National University, Cheongju, Republic of Korea; 2 Department of Family Medicine, Chungbuk National University Hospital, Cheongju, Republic of Korea; 3 Department of Family Medicine, Chungbuk National University College of Medicine, 1 Chungdae-ro, Seowon-gu, Cheongju, 28644 Chungcheongbuk-do Republic of Korea

**Keywords:** Body mass index, Cardiovascular diseases, Cardiometabolic risk factors, Diabetes mellitus, Obesity

## Abstract

**Objective:**

The prevalence of diabetes mellitus (DM), cardio-cerebrovascular diseases (CCVDs) has increased during recent decades. We aimed to investigate the relationship between body mass index (BMI) and each of several outcomes (DM, CCVDs, or mortality) based on the Korean National Health Insurance Service-Health Screening cohort.

**Methods:**

BMI was categorized as appropriate for Asian populations, into underweight (< 18.5 kg/m^2^), normal (18.5–< 23 kg/m^2^), overweight (23–< 25 kg/m^2^), grade 1 obesity (25–< 30 kg/m^2^), grade 2 obesity (30–< 35 kg/m^2^), and grade 3 obesity (≥35 kg/m^2^). In addition, BMI was further stratified into one unit. Multivariate Cox proportional hazards regression analyses were conducted to examine the association between BMI category and the primary outcomes (DM, CCVDs, or mortality).

**Results:**

A total of 311,416 individuals were included. The median follow-up was 12.5 years. Compared to normal BMI, underweight, overweight, and grade 1–3 obese individuals had a higher risk of the primary outcomes (hazard ratio [95% confidence intervals] 1.293 [1.224–1.365], 1.101 [1.073–1.129], 1.320 [1.288–1.353], 1.789 [1.689–1.897], and 2.376 [2.019–2.857], respectively, in men and 1.084 [1.010–1.163], 1.150 [1.116–1.185], 1.385 [1.346–1.425], 1.865 [1.725–2.019], and 2.472 [2.025–3.028], respectively, in women). Setting the reference BMI to 20–< 21 kg/m^2^ and categorizing into one unit increment, BMI was associated with the primary outcomes in a J-shaped manner in both sexes. The risk of DM increased with higher BMI in both sexes, while all-cause mortality decreased in men with a BMI 21–< 31 kg/m^2^ and women with BMI 22–< 30 kg/m^2^.

**Conclusions:**

BMI was associated with all-cause mortality in a J-shaped manner in both sexes, while it was associated with risk of DM in a dose-response relationship. The relationship between BMI and the primary outcomes was J-shaped.

## Introduction

Obesity is defined as excessive adipose tissue. In addition to high caloric intake and low energy expenditure, various etiologies ranging from high glycemic index/load to genetic disorders of appetite control, such as Prader-Willi syndrome, contribute to obesity [1, 2]. Obesity is a modifiable risk factor for non-communicable diseases (NCDs) such as diabetes mellitus (DM), cardio-cerebrovascular diseases (CCVDs), and malignant neoplasms [3–6]. The burden of associated illness has increased during recent decades accompanying the increasing prevalence of obesity [7].

Direct measurements of the amount of fat accumulation in the body are often unavailable and expensive. In clinical practice and population studies, simple anthropometric measurements (body mass index [BMI], waist circumference, waist to hip ratio, etc.) are used to identify obesity. Among them, BMI is the most widely used method to express body size by adjusting weight by squared height, which is approximately the expected value for weight at the given height [8]. The World Health Organization (WHO) and International Obesity Task Force (IOTF) defined overweight and obesity as BMI ≥ 23 kg/m^2^ and ≥ 25 kg/m^2^, respectively, in the Asian population [9]. These cut-off points are lower than those of Western populations because Asian people have more fat content than the white Western people with the same BMI and are more likely to suffer from CCVDs even at lower BMI levels [10]. However, although obesity based on BMI is relatively well correlated with the risk of diabetes and CCVDs, there is a lack of research demonstrating how BMI is associated with the risk of DM, CCVDs, and all-cause mortality in the Korean population.

This study aimed to investigate the relationship between BMI and each outcome (DM, CCVDs, and mortality) using the Korean National Health Insurance Service (NHIS)-Health Screening (HEALS) cohort.

## Methods

### Study population

The Korean NHIS-HEALS cohort consisted of 514,527 individuals randomly selected from the 5.1 million examinees of the Korean national health screening program between 2002 and 2003. The subjects were aged from 40 to 79 years at the end of December 2002. The NHIS-HEALS cohort contains self-reported past medical history, prescription and diagnostic code data based on the national health insurance claim database, death information such as main cause and date of death, and information regarding lifestyle and laboratory tests from national health screening programs.

**Fig. 1:**
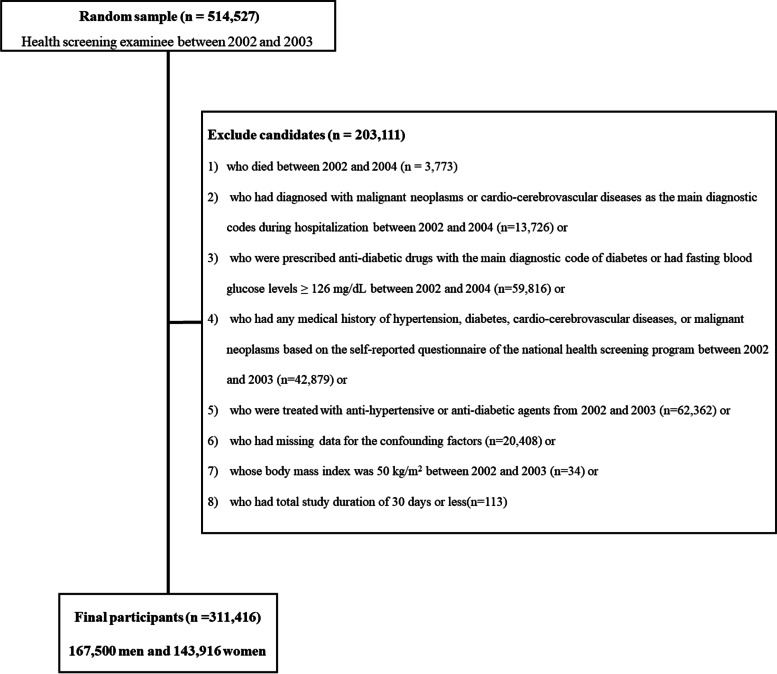
Flowchart of inclusion and exclusion criteria

Figure 1 is a flowchart of the inclusions and exclusions of this study. After exclusion, 311,416 individuals (167,500 men and 143,916 women) were included in this study.

The Institutional Review Board of Chungbuk National University Hospital (CBNUH2021–06-028) approved this study. This research complied with the 1964 Helsinki Declaration. We state to confirm that all methods were carried out in accordance with relevant guidelines and regulations.

### Definition of diabetes, cardio-cerebrovascular diseases, death, and study duration

The study outcomes were the occurrence of diabetes, CCVDs, or death during the study period. DM was defined as the presence of one of the following conditions: 1) a diagnostic code of diabetes (ICD-10 code: E11 – E14) and treatment with any glucose-lowering agents including insulin, sulfonylurea, biguanide, thiazolidinedione, dipeptidyl peptidase-4 inhibitor, alpha-glucosidase inhibitor, sodium-glucose cotransporter-2 inhibitor, and glucagon-like peptide-1 agonist, or 2) a fasting blood glucose of 126 mg/dL or higher on the national health screening program. The onset date of diabetes was set as the earliest of the two dates that satisfied the above conditions. CCVDs (ICD-10 code: cardiovascular diseases, I20 – I25; and cerebrovascular diseases, I60 – I69) were diagnosed when the main diagnosis was recorded twice or more in outpatients or once or more in hospitalized patients. Death was identified by the death certificate. The primary outcome of the study was a composite of DM, CCVDs, or all-cause mortality.

The study start date was defined as the day when a subject participated in the national health screening program. If diabetes, CCVDs, or death occurred, the end date is the earlier date of those events. Otherwise, the end date is the last of either the date of the last outpatient clinic visitation or the last national health check-up.

### Definition of obesity and confounding factors

BMI (unit, kg/m^2^) was calculated by dividing the body weight (kg) by the square of the height (m^2^). BMI was categorized into underweight (< 18.5 kg/m^2^), normal (18.5–< 23 kg/m^2^), overweight (23–< 25 kg/m^2^), grade 1 obesity (25–< 30 kg/m^2^), grade 2 obesity (30–< 35 kg/m^2^), and grade 3 obesity (≥ 35 kg/m^2^) according to the Asia Pacific regional guideline of the WHO and IOTF (9).

Age, systolic blood pressure, lifestyle (tobacco smoking, alcohol consumption, and physical activity), economic status, and laboratory data (glucose and total cholesterol levels) were documented at the baseline exams (2002 and 2003). Lifestyle information and economic status were identified through self-reported questionnaires. Smoking status was categorized into ever smokers (who had smoked cigarettes in the past) and never smokers (who had never smoked cigarettes). Drinking status was divided into rare (drink alcohol less than once a week), sometimes (drink alcohol once to twice a week), and often (drink alcohol three times or more a week). Physical activity was stratified into three groups: rare (engaged in physical exercise for less than one day a week), sometimes (engaged in physical exercise for one to four days a week), and regular (engaged in physical exercise for five or more days a week). Economic status based on the self-reported monthly household income was divided into three groups: low, 0 – 30th percentile, 31st – 70th percentile; and high, 71st – 100th percentile.

### Statistical analysis

Continuous variables were presented as mean ± standard deviation (SD) and categorical variables as number (percentages). To examine the association between BMI category and outcomes, we conducted Cox proportional hazards regression analyses after adjusting for age, systolic blood pressure, glucose, total cholesterol, tobacco smoking, alcohol intake, physical activity, and economic status.

Two-sided tests were considered statistically significant for *p* values less than 0.05. SAS enterprise guide version 7.1 was used as the statistical package (SAS Inc., Cary, NC, USA) and R studio version 3.3.3 was used to conduct the study analyses.

## Results

The median study duration was 12.5 years. Table 1 showed baseline characteristics by sex. The mean age was 50.4 ± 8.6 years for men and 51.4 ± 9.0 for women. Male and female BMI was 23.8 ± 2.8 and 23.5 ± 2.9 kg/m^2^, respectively. The percentage of underweight, normal, overweight, grade 1 obesity, grade 2 obesity, and grade 3 obesity was 2.5, 37.0, 28.4, 30.6, 1.5, and 0.07%, respectively, in men and 2.7, 42.9, 26.2, 25.8, 2.2, and 0.16%, respectively, in women. The percentage of ever smokers was 60.3% in men and 3.6% in women.

**Table 1:** Baseline characteristics according to sex

	Men	Women
Number (N)	167,500	143,916
Age, years	50.4 ± 8.6	51.4 ± 9.0
Body mass index, kg/m^2^	23.8 ± 2.8	23.5 ± 2.9
Systolic blood pressure, mmHg	125.3 ± 15.6	120.5 ± 16.3
Glucose, mg/dL	91.3 ± 12.9	89.5 ± 11.9
Total cholesterol, mg/dL	197.0 ± 36.1	198.1 ± 36.5
Body mass index category, N (%)		
Underweight (< 18.5 kg/m^2^)	4137 (2.5)	3861 (2.7)
Normal (18.5–< 23 kg/m^2^)	61,916 (37.0)	61,708 (42.9)
Overweight (23–< 25 kg/m^2^)	47,585 (28.4)	37,763 (26.2)
Grade 1 Obesity (25–< 30 kg/m^2^)	51,220 (30.6)	37,137 (25.8)
Grade 2 obesity (30–< 35 kg/m^2^)	2531 (1.5)	3220 (2.2)
Grade 3 obesity (≥35 kg/m^2^)	111 (0.07)	227 (0.16)
Smoking status, N (%)		
Never smokers	66,415 (39.7)	138,671 (96.4)
Ever smokers	101,085 (60.3)	5245 (3.6)
Drinking status, N (%)		
Rare	55,825 (33.3)	116,095 (80.7)
Sometimes	79,507 (47.5)	25,058 (17.4)
Often	32,168 (19.2)	2763 (1.9)
Physical activity, N (%)		
Rare	83,575 (49.9)	95,767 (66.5)
Sometimes	69,568 (41.5)	36,091 (25.1)
Regular	14,357 (8.6)	12,058 (8.4)
Economic status, N (%)		
Low	27,488 (16.4)	39,624 (27.5)
Middle	55,054 (32.9)	46,155 (32.1)
High	84,958 (50.7)	58,137 (40.4)

Table 2 presented the results of the Cox proportional hazards regression model to investigate the association between BMI category and incidence of diabetes, CCVDs, and all-cause mortality after adjusting for age, systolic blood pressure, glucose, total cholesterol, tobacco smoking, alcohol intake, physical activity, and economic status. In this table, BMI category was classified according to the Asia Pacific regional guideline of the WHO and IOTF. Compared to normal BMI, hazard ratios (HRs) (95% confidence intervals [CIs]) for diabetes of underweight, overweight, and grade 1 to 3 obesity were 0.843 (0.717–0.990), 1.603 (1.524–1.685), 2.431 (2.321–2.546), 4.784 (4.371–5.237), and 5.592 (3.845–6.624) respectively, in men and 0.744 (0.607–0.911), 1.624 (1.528–1.725), 2.567 (2.43–2.712), 4.372 (3.991–4.788), and 4.808 (3.624–6.378), respectively, in women. After being fully adjusted, HRs (95% CIs) for CCVDs of underweight, overweight, and grade 1 to 3 obesity were 0.974 (0.894–1.060), 1.109 (1.073–1.146), 1.200 (1.162–1.241), 1.351 (1.226–1.489), and 1.002 (0.551–1.821), respectively, in men and 0.907 (0.827–0.995), 1.099 (1.060–1.140), 1.169 (1.128–1.211), 1.264 (1.158–1.380), and 1.462 (1.101–2.215) respectively, in women. In contrast to diabetes and CCVDs, HRs (95% CIs) for all-cause mortality of overweight and grade 1 obesity decreased. Those for underweight, overweight, and grade 1 to 3 obesity were 1.710 (1.596–1.832), 0.780 (0.744–0.817), 0.730 (0.694–0.767), 0.948 (0.807–1.114), and 1.029 (0.613–1.863), respectively, in men and 1.698 (1.524–1.893), 0.801 (0.744–0.862), 0.830 (0.773–0.891), 1.123 (0.949–1.331), and 1.368 (0.869–2.306), respectively, in women. HRs (95% CIs) for the primary outcomes (DM, CCVDs, or all-cause mortality) of underweight, overweight, and grade 1 to 3 obesity were 1.293 (1.224–1.365), 1.101 (1.073–1.129), 1.320 (1.288–1.353), 1.789 (1.689–1.897), and 2.376 (2.019–2.857), respectively, in men and 1.084 (1.010–1.163), 1.150 (1.116–1.185), 1.385 (1.346–1.425), 1.865 (1.725–2.019), and 2.472 (2.025–3.028), respectively, in women.

**Table 2:** Cox-proportional hazards regression model for the incidence of diabetes, cardio-cerebrovascular diseases, or all-cause mortality according to body mass index category

		Underweight (BMI < 18.5 kg/m^2^)	Normal (18.5–< 23 kg/m^2^)	Overweight (23–< 25 kg/m^2^)	Grade 1 Obesity (25–< 30 kg/m^2^)	Grade 2 Obesity (30–< 35 kg/m^2^)	Grade 2 Obesity (≥35 kg/m^2^)
**Primary Outcomes**^**a**^	**Men**	1.293 (1.224–1.365)	1	1.101 (1.073–1.129)	1.320 (1.288–1.353)	1.789 (1.689–1.897)	2.376 (2.019–2.857)
	**Women**	1.084 (1.010–1.163)	1	1.150 (1.116–1.185)	1.385 (1.346–1.425)	1.865 (1.725–2.019)	2.472 (2.025–3.028)
**Diabetes**	**Men**	0.843 (0.717–0.990)	1	1.603 (1.524–1.685)	2.431 (2.321–2.546)	4.784 (4.371–5.237)	5.592 (3.845–6.624)
	**Women**	0.744 (0.607–0.911)	1	1.624 (1.528–1.725)	2.567 (2.43–2.712)	4.372 (3.991–4.788)	4.808 (3.624–6.378)
**CCVDs**	**Men**	0.974 (0.894–1.060)	1	1.109 (1.073–1.146)	1.200 (1.162–1.241)	1.351 (1.226–1.489)	1.002 (0.551–1.821)
	**Women**	0.907 (0.827–0.995)	1	1.099 (1.060–1.140)	1.169 (1.128–1.211)	1.264 (1.158–1.380)	1.462 (1.101–2.215)
**All-cause mortality**	**Men**	1.710 (1.596–1.832)	1	0.780 (0.744–0.817)	0.730 (0.694–0.767)	0.948 (0.807–1.114)	1.029 (0.613–1.863)
	**Women**	1.698 (1.524–1.893)	1	0.801 (0.744–0.862)	0.830 (0.773–0.891)	1.123 (0.949–1.331)	1.368 (0.869–2.306)

Adjusted for age, systolic blood pressure, glucose, total cholesterol, tobacco smoking, alcohol intake, physical activity, and economic status

*Abbreviation: CCVDs* Cardio-cerebrovascular diseases

^a^Primary outcomes include diabetes mellitus, cardio-cerebrovascular diseases, or all-cause mortality

**Fig. 2:**
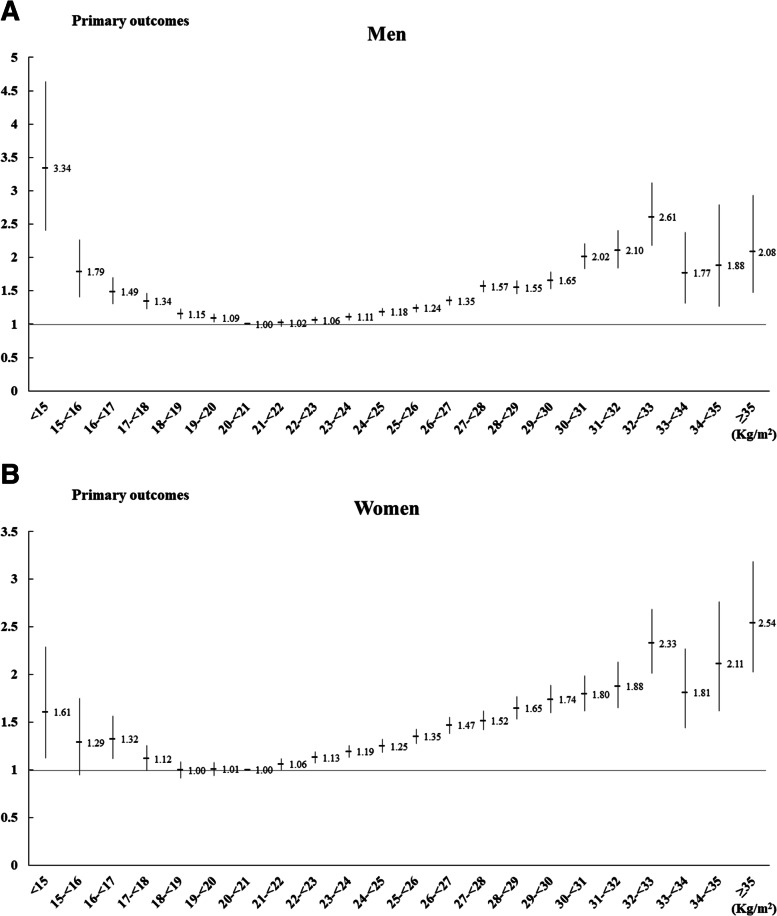
Cox-proportional hazards regression models for the primary outcomes (diabetes mellitus, cardio-cerebrovascular diseases, or all-cause mortality) according to body mass index. Adjusted for age, systolic blood pressure, glucose, total cholesterol, tobacco smoking, alcohol intake, physical activity, and economic status

Figure 2 showed the Cox proportional hazards regression models for incidence of the primary outcomes (DM, CCVDs, or all-cause mortality) according to BMI after adjusted for age, systolic blood pressure, glucose, total cholesterol, tobacco smoking, alcohol intake, physical activity, and economic status. BMI of 20–< 21 kg/m^2^ was set at 1.0 as the reference value. In Fig. 2, a J-shaped relationship was observed in both sexes between BMI and the incident primary outcomes. The HRs for the incidence of the primary outcomes significantly increased from BMI < 20 kg/m^2^ for men while they increased from BMI < 17 kg/m^2^ for women. In addition, BMI ≥22 kg/m^2^ for men and ≥ 21 kg/m^2^ for women significantly elevated the risk for incidence of DM, CCVDs, or all-cause mortality.

**Fig. 3:**
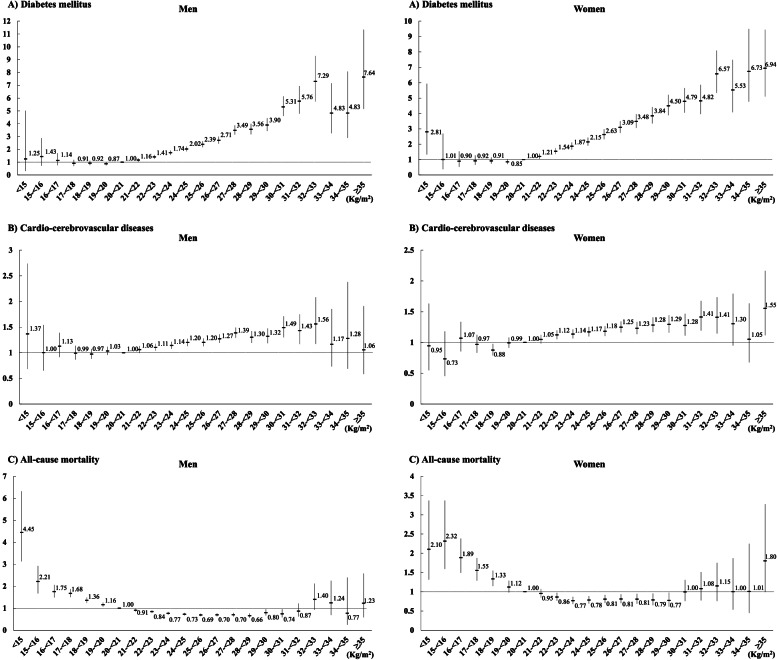
Cox-proportional hazards regression models for the risk of diabetes mellitus, cardio-cerebrovascular diseases, and all-cause mortality according to body mass index. **A** Diabetes mellitus. **B** Cardio-cerebrovascular diseases. **C** All-cause mortality. Adjusted for age, systolic blood pressure, glucose, total cholesterol, tobacco smoking, alcohol intake, physical activity, and economic status

Figure 3 presented the relationship between BMI and each outcome (DM, CCVDs, and all-cause mortality). The risk of DM linearly increased with higher BMI in both sexes, as seen in Fig. 3A. Compared with BMI between 20 and 21 kg/m^2^, the risk of CCVDs was higher in individuals with BMI between 22 and 33 kg/m^2^ in both sexes. Figure 3C demonstrated the relationship between BMI and all-cause mortality. The HRs for the risk of all-cause mortality were significantly high in BMI less than 20 kg/m^2^ in men and 19 kg/m^2^ in women, while they decreased for BMI between 21 and 31 kg/m^2^ in men and 22 and 30 kg/m^2^ in women.

## Discussion

In this study, BMI was associated with the primary outcomes (DM, CCVDs, or all-cause mortality) in a J-shaped manner in both sexes, while the risk of diabetes increased linearly with BMI. Underweight had the highest risk of all-cause mortality while overweight and grade 1 obesity were negatively associated. These relationships showed similar patterns whether using the conventional obesity categories or performing statistical analyses by one unit of BMI.

Although the prevalence of obesity have stabilized in Korea [11], its worldwide prevalence is increasing [12]. Obesity is positively associated with high risk of diabetes and CCVDs [3, 4]. However, a systematic review and meta-analysis by Flegal et al. demonstrated that obesity has a J-shaped association with all-cause mortality [13]. According to Flegal’s study, the association between BMI category and all-cause mortality was significantly different: overweight (BMI, 25–< 30 kg/m^2^) reduced, grade 1 obesity (BMI, 30–< 35 kg/m^2^) did not change, and grade 2 obesity or more (BMI ≥35 kg/m^2^) increased all-cause mortality [13]. Our study is in part consistent with previous studies [3, 4]. The incidence of DM and CCVDs increased with higher BMI. However, overweight (BMI, 23–< 25 kg/m^2^) and grade 1 obesity (BMI, 25–< 30 kg/m^2^) decreased all-cause mortality and grade 2 obesity (BMI, 30–< 35 kg/m2) was not significantly elevated. Recently, Bae et al. suggested that the cut-off points of BMI should be differently defined depending on the disease [6]. They reported a J-shaped association between BMI and the incidence of DM and CCVDs. However, the current study demonstrated that the risk of DM and to a lesser extent CCVDs seems to linearly increase with increasing BMI. This discrepancy might be due to the different study population. Bae et al.’s study included 8900 individuals from the Korean Genome and Epidemiology Study, while this study included a larger population after excluding individuals with a past or current medical history of malignant neoplasms, DM, hypertension, dyslipidemia, and CCVDs. These exclusion criteria can minimize the effect of pre-existing confounding factors. Another Korean study by Hong et al. demonstrated an L-shaped relationship between BMI and the risk of myocardial infarction, stroke, and all-cause mortality in the Korean elderly population [14]. Peculiarly, the higher BMI categories corresponding to overweight and obesity reduced the incidence of myocardial infarction and stroke, as well as all-cause mortality. Because their study of an elderly population did not exclude for past medical history of myocardial infarction and stroke, the findings of our study including middle-aged or older individuals may be different from theirs.

This current study is consistent with previous studies reporting the “obesity paradox” [15, 16]. The obesity paradox is that overweight or obesity are paradoxically associated with lower or not higher risk of mortality [15–17]. The possible mechanisms leading to the obesity paradox were suggested as follows: survival effect; time discrepancy of competitive risk factors; reverse causation; prevention or delay in cognitive decline; protection from osteoporotic fracture due to reduced bone mineral loss; and energy reserve and prevention of malnutrition [17]. This study is meaningful in proving that the obesity paradox occurs even in relatively young and healthy people. However, contrary to the inverse association between BMI and all-cause mortality, DM and CCVDs risk increased directly with BMI. These results are in agreement with the well-known association of DM and CCVDs with obesity [3, 4]. The J-shaped relationship between BMI and the incident primary outcomes (DM, CCVDs, or all-cause mortality) did not differ by gender. This J-shaped relationship supports that the BMI cutoff points assigned by WHO and IOTF Asia Pacific regional guidelines is appropriate for healthy Korean adults [9].

This study has several limitations and should be interpreted cautiously. First, we used BMI to define general underweight, overweight, and obesity. Although BMI is well correlated with body fatness [18], it does not distinguish fat from fat-free mass and can overestimate adiposity in muscular subjects. BMI has been well validated with body fat percentage as assessed by dual X-ray absorptiometry and can be clinically useful [19], but lean body mass or lean body mass index might be a better indicator to predict future mortality [19, 20]. However, data regarding lean body mass were not collected in this study. Second, caloric intake and nutritional status were not measured and controlled in this study. Caloric intake and nutrition are closely associated with all-cause mortality and the risk of DM and CCVDs [21, 22]. According to Livingstone’s study, better dietary quality reduced mortality due to all-cause and CCVDs but proinflammatory diets increased mortality [22]. If information regarding diet and nutritional status was available, more robust results could have been derived. Third, it is possible that there were underlying diseases that had not been diagnosed at baseline. In order to reduce bias, individuals whose study period was less than 30 days were excluded, but this may be insufficient. Fourth, it seems that the associations between BMI and health outcomes were distorted because the number of individuals with BMI ≥30 kg/m^2^ was too small. Two thousand six hundred forty-two men and 3447 women who belong to grade 2 obesity or higher consisted of only 1.6 and 2.4% of the total study population, respectively. This disproportional number may have affected the results.

Despite these potential limitations, this study has several advantages. A large number of subjects (311,416 individuals) were followed for a long period (median follow-up duration 12.5 years). Individuals with pre-existing malignant neoplasms, CCVDs, and DM were excluded at baseline. These exclusions may help minimize bias introduced by pre-existing diseases or risk factors. In addition to the primary outcomes, the relationship between BMI and each outcome (DM, CCVDs, and all-cause mortality) was investigated after single unit stratification of BMI.

In conclusion, a J-shaped relationships were observed between BMI and the primary outcomes. BMI was linearly associated with risk of developing of DM and to a lesser extent CCVDs. Consistent with an obesity paradox, all-cause mortality for baseline overweight and grade 1 obesity was lower than was observed for individuals categorized normal weight by BMI.

## Data Availability

The data that support the findings of this study are available from the Korean National Health Insurance Database but restrictions apply to the availability of these data, which were used under license for the current study, and so are not publicly available. Data are however available from the corresponding author upon reasonable request and with permission of the Korean National Health Insurance Database.

## Abbreviations

BMI: Body mass index
CCVD: Cardio-cerebrovascular disease
CI: Confidence interval
DM: Diabetes mellitus
ICD: International classification of disease
IOTF: International obesity task force
HEALS: Health Screening
HR: Hazard ratio
NCD: Non-communicable disease
NHIS: National health insurance service
WHO: World health organization
